# Insights into the influence of physicochemical parameters on the microbial community and volatile compounds during the ultra-long fermentation of compound-flavor Baijiu

**DOI:** 10.3389/fmicb.2023.1272559

**Published:** 2023-10-26

**Authors:** Wei Cheng, Xuefeng Chen, Wei Lan, Gengdian Liu, Xijia Xue, Ruilong Li, Tianquan Pan, Na Li, Duan Zhou, Xingjie Chen

**Affiliations:** ^1^School of Food Science and Engineering, Shaanxi University of Science & Technology, Xi’an, China; ^2^Technology Center of Enterprise, Jinzhongzi Distillery Co., Ltd., Fuyang, China; ^3^School of Biology and Food Engineering, Fuyang Normal University, Fuyang, China

**Keywords:** compound-flavor Baijiu, fermented grains, ultra-long fermentation, amplicon sequencing, volatile compounds

## Abstract

**Introduction:**

While the variation in physicochemical parameters, microbial communities, metabolism, composition, and the proportion of volatile components in fermented grains (FG) affect final Baijiu quality, their complex interactions during the ultra-long fermentation of compound-flavor Baijiu (CFB) are still poorly understood.

**Methods:**

In this study, amplicon sequencing was used to analyze the microbial community, and headspace solid-phase microextraction-gas chromatography–mass spectrometry (HS-SPME-GC–MS) was used to analyze the volatile components in FG during ultra-long fermentation of CFB. The relationships between the dominant microbial communities, physicochemical parameters, and volatile components were analyzed using redundancy analysis and network analysis.

**Results:**

During ultra-long fermentation, bacterial diversity was initially higher than during the mid and late stages. Fungal diversity in the mid stages was higher than that initially and later in the process. A total of 88 volatile components, including six alcohols, 43 esters, eight aldehydes and ketones, 13 acids, and 18 other compounds were detected in FG. Starch and reducing sugars in FG strongly affected the composition and function of bacterial and fungal communities. However, acidity had little effect on the composition and function of the bacterial flora. *Lactobacillus, Bacillus, Weissella*, and *Pichia* were the core microbial genera involved in metabolizing the volatile components of FG.

**Discussion:**

We provide insights into the relationships and influences among the dominant microbial communities, physicochemical parameters, and volatile components during ultra-long fermentation of CFB. These insights help clarify the fermentation mechanisms of solid-state fermentation Baijiu (SFB) and control and improve the aroma quality of CFB.

## 1. Introduction

Chinese Baijiu is produced using sorghum as the main raw material and Daqu, Xiaoqu, and Fuqu as saccharation starter cultures via complex enzymatic chemical reactions, biochemical reactions, and microbial metabolic activities (Xu et al., 2017). Fermented grains (FG) are carriers for microbial fermentation and direct sources of aroma substances. Their microbial communities and succession have a strong influence on the aroma components of Baijiu (Jin et al., 2017; Xu et al., 2022). During the brewing process, the physicochemical parameters of FG affect microbial communities and their metabolism. This leads to changes in the composition and proportion of flavor components, which affect the quality of Baijiu (Xiang et al., 2013; Guan et al., 2020). Alcohol and water are the principal components of Baijiu, and the quality of Baijiu mainly depends on the concentration of other volatile compounds, which including esters, higher alcohols, acids, and phenols (Li et al., 2014). The generation of these volatile components are mainly influenced by the microbial succession or produced by microbial metabolism during the brewing process of fermented grains. In addition, physicochemical parameters of fermented grains have important effects on the succession and metabolism of microbial (Liu et al., 2021). Therefore, it is important to clear the relationships of physicochemical parameters, microbial community and volatile components in FG during fermentation to determine the fermentation mechanism of FG and improve Baijiu quality.

Compound-flavor Baijiu (CFB) is derived from two or more of the four main flavor styles (Xiao et al., 2016; Cheng et al., 2022). The fermentation time of CFB is approximately 60–80 d (Cheng et al., 2022), and the normal fermentation time is 60 days. Owing to the influence of climatic factors, brewing workshops usually stop production in summer in central and southern China, leading to an ultra-long fermentation time for grains, with fermentation of approximately 180 d. Several studies have investigated physicochemical parameters, microbial communities, and aroma components during the brewing process of FG (Wang et al., 2018, 2021; Zhang et al., 2007, 2021). However, data on the relationships between physicochemical parameters, microbial communities, and volatile components of FG during ultra-long fermentation processes of CFB remain limited, specifically with respect to the major producing regions of the famous Jianghuai Baijiu in China. And so far, there are almost no research on the effects of ultra-long fermentation time on microbial composition, physicochemical parameters, and flavor components in FG of CFB.

In this study, we analyzed the physicochemical parameters, microbial community, and volatile components of FG to clarify the correlations between these factors. The results of this study help guide the adjustment of the physicochemical parameters of FG, determine the fermentation mechanism, and provide a basis for optimizing the brewing process of CFB to improve its quality.

## 2. Materials and methods

### 2.1. Sample collection

FG samples were collected from the brewing workshop of Anhui Jinzhongzi Distillery Co., Ltd. During ultra-long fermentation, early stage FG at 1, 5, 10, and 20 d, middle stage FG at 30, 45, and 60 d, and late stage FG at 90, 120, 150, and 180 d were sampled and termed AF1, AF5, AF10, AF20, AM30, AM45, AM60, AE90, AE120, AE150, and AE180.

Each sample was collected from the upper, middle, and lower layers of the fermentation cellar, before being sampled at three points in each layer and then mixed (Supplementary Figure S1). Three parallel samples were randomly collected, mixed, divided into two parts, and transferred into two bags. One bag was stored at −80°C for DNA extraction, and the other bag was stored at 4°C for the detection of physicochemical parameters and volatile compounds.

### 2.2. Determining physicochemical parameters and temperature

The gravimetric method was used to determine the moisture of FG, and the samples were dried at 105°C for at least 3 h to constant weight. The titratable acidity, starch, and sugar contents of FG were reduced (Wang et al., 2021). A cellular digital display thermometer was inserted into the central sampling point (Supplementary Figure S1B), and the temperature was recorded.

### 2.3. Extraction and sequencing of the total DNA of FG

Sample pretreatment was performed according to the method described by Wang et al. (2021) based on the operation instructions of the EZNA Soil DNA Kit (Omega Bio-tek, Norcross, GA, United States) to extract total DNA. Diluted genomic DNA was used as a template, and specific primers with barcodes were used according to the selection of the sequencing region and high efficiency Hi-fi enzymes for PCR to ensure amplification accuracy. For bacteria, the forward primer 515F (GTGYCAGCMGCCGCGGTAA) and reverse primer 806R (GGACTACNVGGGTWTCTAAT) were used to amplify the 16S V3–V4 (b) domain. Moreover, ITS5-1737F (GGAAGTAAAAGTCGTAACAAGG) and ITS2-2043R (GCTGCGTTCTTCATCGATGC) were used to amplify the fungal ITS1 (a) sequence region.

The QuantiFluor-ST Blue Fluorescence Quantitative System (Promega, Madison, WI, United States) was used to assess the concentration of the polymerase chain reaction products. The total genomic DNA was sequenced on a MiSeq Benchtop Sequencer (2 × 300 bp; Illumina MiSeqPE300, San Diego, CA, United States) by BioYigene Biotechnology Co., LTD (Wuhan, China).

### 2.4. Processing raw sequencing data

After completing high-throughput sequencing, the QIIME2 (2019.4) software was used to optimize the quality of the sequencing data. Sequences with a similarity greater than or equal to 97% were classified into the same OTU. All sequences were compared with the Silva (Silva_136.1) database to obtain taxonomic information. A community composition map and heatmap were drawn using R on the Illumina MiSeq sequencing platform.

### 2.5. Analysis of volatile components in FG using HS-SPME-GC–MS

Pre-treatment of samples was conducted as previously described, with adjustments (Hu et al., 2021; Yan et al., 2021). A total of 10 g of FG were weighed and mixed with 1.0% CaCl_2_, 20 mL of boiled ultra-pure water, and soaked overnight at 4°C. The mixture was then ultrasonicated in an ice water bath for 30 min, centrifuged at 10,000 rpm at 4°C for 20 min, and 5 mL of the supernatant was placed in a 20 mL headspace bottle with 3 g of NaCl and 20 μL of amyl acetate (internal standard, concentration 0.3612 g L^−1^).

A three-phase extraction head (DVB/CAR/PDMS, 50/30 μm) with an initial temperature of 50°C, preheating for 5 min, extraction for 45 min, and desorption for 5 min was used. The column length, inner diameter, and liquid film thickness of the DB-FFAP chromatographic column were 60 m, 0.25 mm, and 0.25 μm, respectively. The GC gradient conditions were as follows: the initial temperature was 50°C for 2 min, and then, the temperature was increased at 6°C min^−1^ to 230°C for 15 min. The temperature of the inlet and the detector were 250°C, the carrier gas was high purity helium (He); the flow rate was 2 mL min^−1^, not shunt. The junction temperature of the MS was 250°C, the quadrupole temperature was 150°C, and the scanning range was 35–350 amu.

The National Institute of Standards and Technology database (NIST 05 s) was used to match unknown compounds. A matching degree greater than 80% was selected as the threshold and combined with the retention time of C_7_–C_40_ to calculated retention index (RI), and compared it with the RI reported on the NIST website to qualitative analysis of unknown compounds. In addition, RI = 100 Z + 100 [TR(*x*) − TR(*z*)]/[TR(*z* + 1) − TR(*z*)], according to the reference (Huang et al., 2022). The relative signal intensity of amyl acetate was used to calculate the percentage area of each peak, and the concentration of each substance was calculated.

### 2.6. Data processing and drawing of network correlation diagram

Statistical analyses were performed using the Statistical Package SPSS (version 21.0). Spearman correlation coefficients between the top-ten dominant microorganisms and 88 volatile components were calculated to analyze the relationships between microbial communities and volatile compounds. The vegan and heatmap packages in R (version 3.2.4) and the vegan package (version 2.3–4) were used (Hao et al., 2021). Based on physicochemical parameters, volatile compounds, and the relative abundance of bacterial and fungal communities, redundancy analysis (RDA) was used. A correlation heatmap was then created. For the data with absolute coefficients greater than 0.5 and significant correlation, a network correlation analysis was created using Cytoscape 3.4.0.

## 3. Results and discussion

### 3.1. Physicochemical parameters and temperature changes of FG during ultra-long fermentation

The physicochemical parameters of FG, that is, moisture, acidity, reducing sugar, and starch are key factors affecting the microbial community structure. Physicochemical parameters are also affected by microbial metabolism (Shen et al., 2021; Wang et al., 2021). The FG temperature directly affected the variation in microbial species and generation and accumulation of metabolites (Hao et al., 2021).

The starch and reducing sugars in FG decreased during the ultra-long fermentation process. This decrease was greater during the early fermentation period (Supplementary Figure S2A). It was potentially affected by the growth and metabolism of molds in the early brewing period. Molds can produce a substantial amount of amylase to hydrolyze starch and produce monosaccharides. This can affect the microbial flora during the brewing process (Liu and Sun, 2018). The acidity of FG increased during the ultra-long fermentation process (Supplementary Figure S2A), which was related to the proliferation of anaerobic and facultative anaerobic bacteria that produce many organic acids (Hao et al., 2021). The acidity of FG is predominantly derived from the metabolism of the organic acids of acid-producing bacteria. These could be used as the main flavor compounds in Baijiu and are regarded as precursor substances for the formation of esters (Liu and Sun, 2018; Wang et al., 2018). Comparing with the normal fermentation (60 days), the starch and reducing sugars in FG keep small range of fluctuations during the ultra-long fermentation process (180 days). However, the acidity in FG still keeps increasing after the normal fermentation.

During the ultra-long fermentation process, the temperature of FG reached 38°C on the 10th day and remained above 38°C for approximately 20 d. Subsequently, the FG temperature decreased slightly and remained unchanged at the later stage (Supplementary Figure S2B). Initially, the starch and reducing sugar contents in FG were relatively high. The rapid propagation of microorganisms led to the generation of fermentation heat, which may have led to an increase in temperature.

### 3.2. Analysis of FG microbial community structure during the ultra-long fermentation process

#### 3.2.1. Venn analysis of sequence statistics, alpha diversity, and OTU distribution

**Figure 1:**
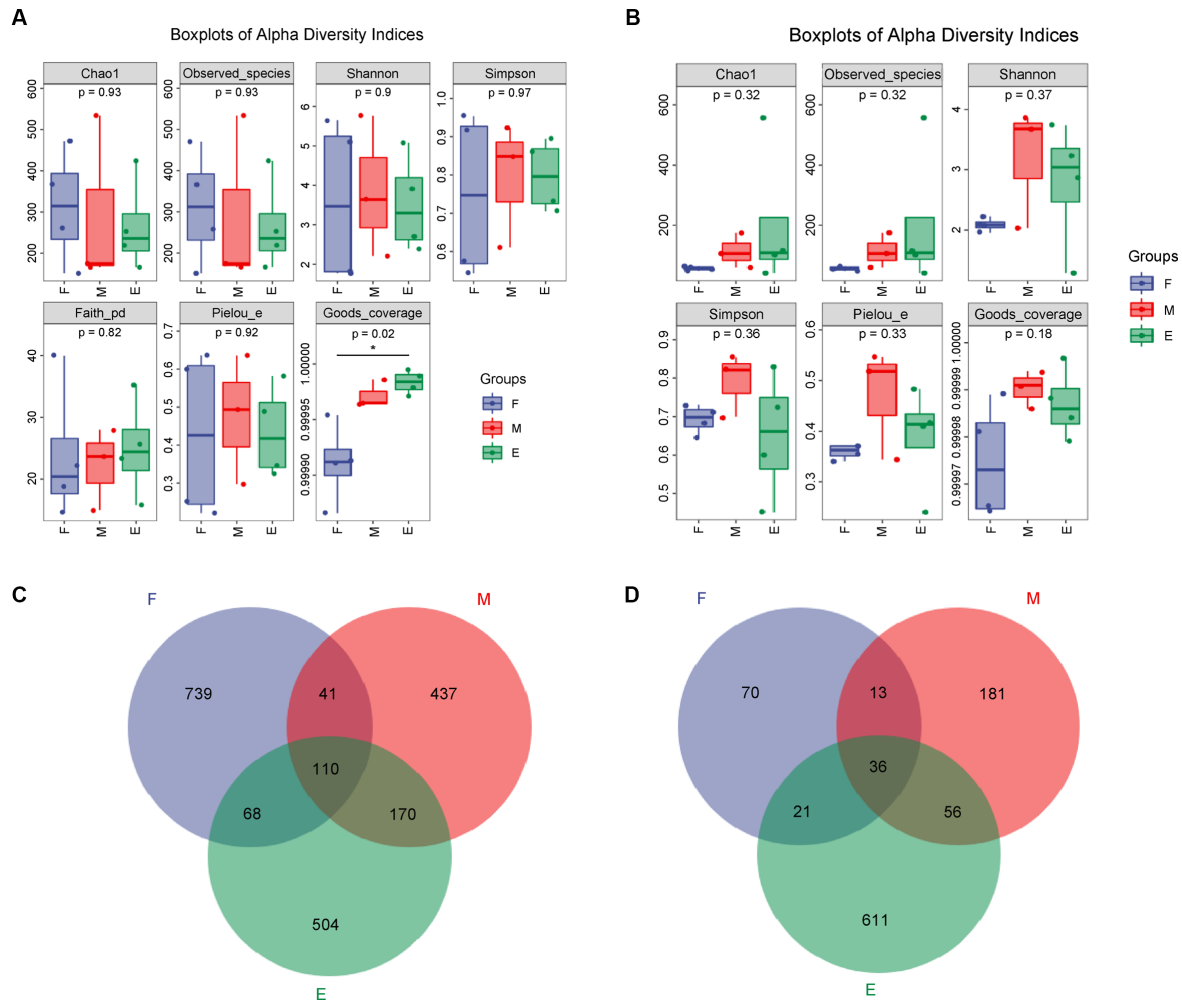
Analysis of microbial alpha diversity of FG during the ultra-long fermentation process. **(A)** Alpha diversity of bacteria, **(B)** alpha diversity of fungal, **(C)** Venn of microbial OTU of bacteria, **(D)** Venn of microbial OTU of fungal.

After quality control filtration, 103, 078 ~ 140, 822 bacterial sequences were obtained with an average length of 162–376 bp. In total, 3,204 OTUs were generated using cluster analysis. In addition, 93, 715 ~ 121, 901 fungal sequences were obtained, with an average length of 105–396 bp. A total of 1,634 OTUs were generated using cluster analysis. The coverage of samples in each group was greater than 0.999, and *p*-values of the Chao1 index were 0.93 and 0.32 (Figure 1). Abundance and diversity of microbial communities can be reflected by alpha diversity (Schloss et al., 2011). The dilution curve, Shannon curve, and alpha diversity indices (Supplementary Figure S3 and Supplementary Tables S1, S2) indicated that the metagenomic sequencing data obtained were representative of the samples.

The Chao1 index of the bacteria in Group F was the highest among the groups (Figure 1A). The Chao1 index of fungi, indicating fungal richness, in Group E was the highest (Figure 1B). The number, evenness, and diversity of species in the samples are indicated by the Shannon and Simpson indices (Wang et al., 2018). The Shannon and Simpson indices for bacteria in Group F were the highest (Figure 1A), while these indices for fungi were both the highest in Group M (Figure 1B). In conclusion, the bacterial diversity of FG in the early stage of ultra-long fermentation was higher than that in the middle and later stages. The fungal diversity in the middle stage was higher than that in the early and late stages.

There were 110 bacterial OTUs in the different groups, accounting for 5.32% of total bacterial OTUs. The maximum number of bacterial OTUs (958) was highest in Group F, accounting for 46.30% of the total bacterial OTUs in the groups, indicating that Group F had the highest bacterial diversity (Figure 1C). There were 36 fungal OTUs in the different groups, accounting for 3.64% of the total fungal OTUs (Figure 1D). The maximum number of fungal OTUs in Group E was 724, accounting for 73.28% of the total fungal OTUs, indicating that Group E had the highest fungal diversity. The fungal OTU number in Group M was higher than that in Groups F and E. This indicated that the diversity of fungi in the FG at the middle stage was the highest among the different stages of the brewing process, which was consistent with the results of the Shannon and Simpson indices.

#### 3.2.2. Microbial community structure and linear discriminant analysis effect size of FG

**Figure 2:**
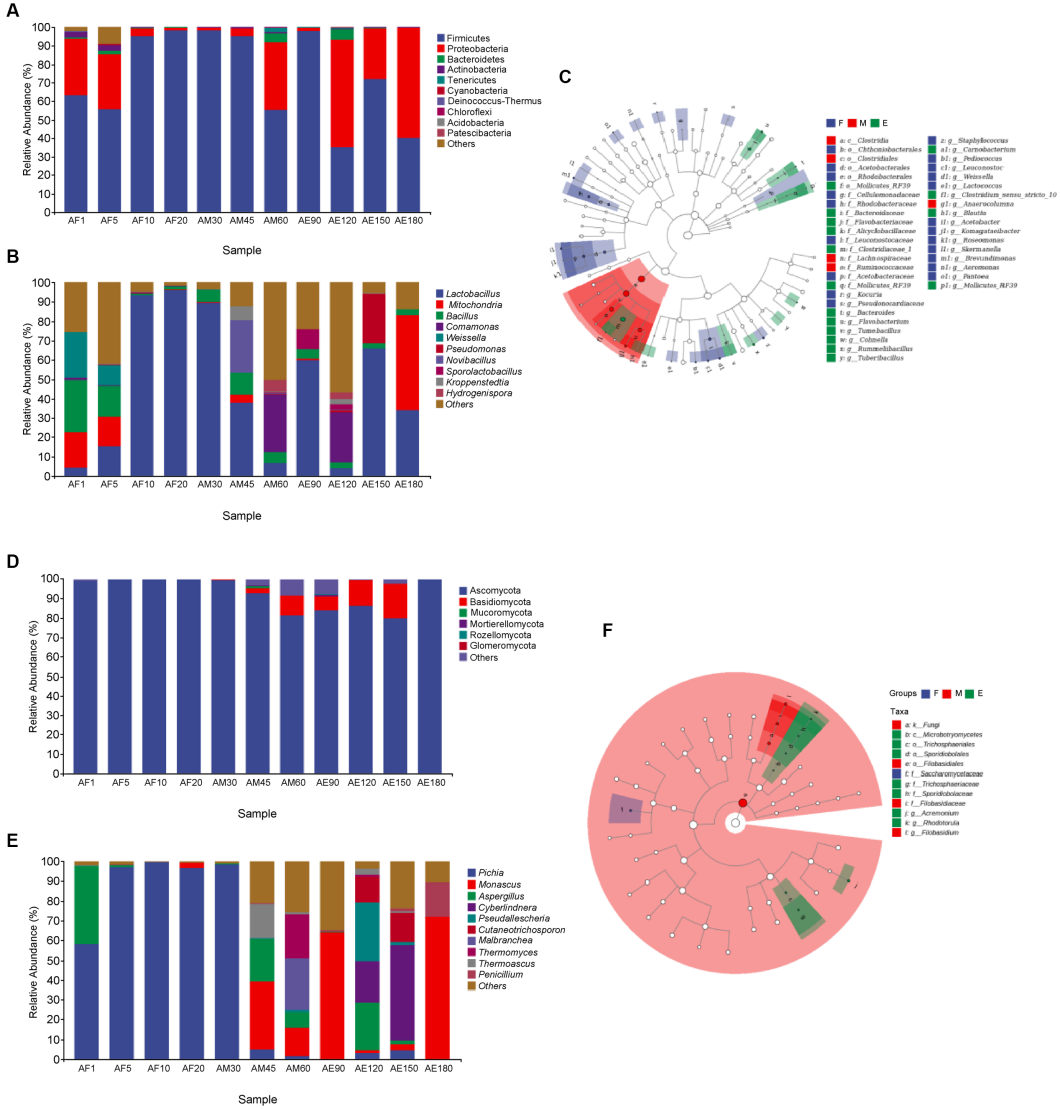
Microbial community structure in FG during the ultra-long fermentation process. **(A)** Bacterial on phylum level, **(B)** bacterial on genus level, **(C)** Lefse analysis of differential species annotated clade of bacterial, **(D)** fungal on phylum level, **(E)** fungal on genus level, and **(F)** Lefse analysis of differential species annotated clade of fungal.

As shown in Figure 2A, the bacteria in FG could be classified into 13 phyla. The dominant phylum in FG was *Firmicutes*, and its relative abundance in different samples was more than 30.00%. In sample AE180, the relative abundances of *Firmicutes* and *Proteobacteria* were 39.82 and 59.73%. As shown in Figure 2D, the fungi in FG could be classified into six phyla. *Ascomycota* in FG was the dominant phylum, and its relative abundance in different samples was higher than 80.00%. At the end of ultra-long fermentation, the relative abundance of *Ascomycota* was 99.93%. *Ascomycota* is a key phylum that plays an important role in brewing different Baijiu styles, such as soy sauce (Song et al., 2017) and Luzhou-flavored Baijiu (Zhang et al., 2021). Comparing with the normal fermentation (60 days), the dominant phylum of bacteria in FG was changed to *Firmicutes* and *Proteobacteria* instand of *Firmicutes*, the dominant phylum of fungi changed to *Ascomycota* instand of *Ascomycota* and *Basidiomycota* during the ultra-long fermentation process (180 days). It reported that *Ascomycota* is the main fungus in FG of sauce-flavored, strong-flavored, and light-flavored baijiu, indicating this phylum is one of the key fungal microflora in the brewing of baijiu (Luo et al., 2023).

As shown in Figure 2B, the bacteria in FG can be classified into four main genera: *Lactobacillus*, *Mitochondria*, *Bacillus*, and *Comamonas*. The relative abundance of *Lactobacillus* in AF10, AF20, AM30, and AM45 was higher than 35.00%, and *Lactobacillus* was the dominant bacteria in FG. Lactic acid is an important flavor substance, which is helpful in increasing the thickness and reducing the irritability of Baijiu, and *Lactobacillus* is an important lactic acid-producing bacterium in FG (Wang et al., 2017). It is likely that the lactic acid in FG is mainly produced during the middle and late stages of the ultra-long fermentation period. The relative abundance of *Bacillus* was higher than 2.50% during the late stage of ultra-long fermentation. *Bacillus* has high temperature resistance, enzyme production, and fragrance production, and its metabolites include pyrazines, acids, methyl esters, and other flavor substances (Li et al., 2014). Comparing with the normal fermentation (60 days), the dominant genera of bacteria in FG was changed to *Mitochondria* and *Lactobacillus* instand of *Comamonas* and *Lactobacillus*. In addition, the dominant genera of fungi changed to *Monascus* and *Penicillium* instand of *Malbranchea*, *Thermomyces*, and *Monascus* during the ultra-long fermentation process (180 days).

As shown in Figure 2E, the dominant genera in the early and middle stages were *Pichia* (AF1, AF5, AF10, AF20, and AM30), and their relative abundances were higher than 58.00%. The relative abundance of *Pichia* was low during the middle (AM45, AM60, and AM90) and late stages of fermentation. *Wickerhamomyces* and *Pichia* play major roles in aroma production and metabolism during Baijiu fermentation (Wang et al., 2019). The relative abundance of *Monascu* was 72.04% in the later stages of ultra-long fermentation, indicating that *Monascu* was the dominant fungal genus. *Monascus* is a unique functional fungus that appears in FG and plays an important role in promoting the formation of esters during the fermentation of Baijiu (Xu et al., 2021, 2022).

Linear discriminant analysis effect size (LEfSe) is helpful in understanding the differences in microflora and microbial species between groups. As shown in Figures 2C,F, 29 bacterial genera were upregulated in the early stages, including *Kocuria*, *Pantoea*, *Saccharopolyspora_virgula*. Five bacterial and four fungal genera were upregulated during the middle stage. At the later stage, *Bacteroides*, *Flavobacteriacea*, and *Mollicutes_RF39* were among the 16 upregulated bacterial genera. Among the seven fungal genera, *Sporidiobolaceae*, *Rhodotorula*, and *Sporidiobolales* were upregulated.

Tang et al. (2022) reported that the acidity and alcohol content increased followed the extension of fermentation time, which led to the death of aerobic microbiota, such as molds and most types of yeasts, but survival of anaerobes and facultative anaerobic microorganisms. In our study, amplicon sequencing was used and mainly analyzed the relative abundance of microbial community in different samples of FG at the genus level. Further, the strength of the results should be validated by the culture-dependent analysis, and we are planning to tackle these questions in future work.

#### 3.2.3. Heatmap and network correlation analysis of FG microbial community structure at the genus level

**Figure 3:**
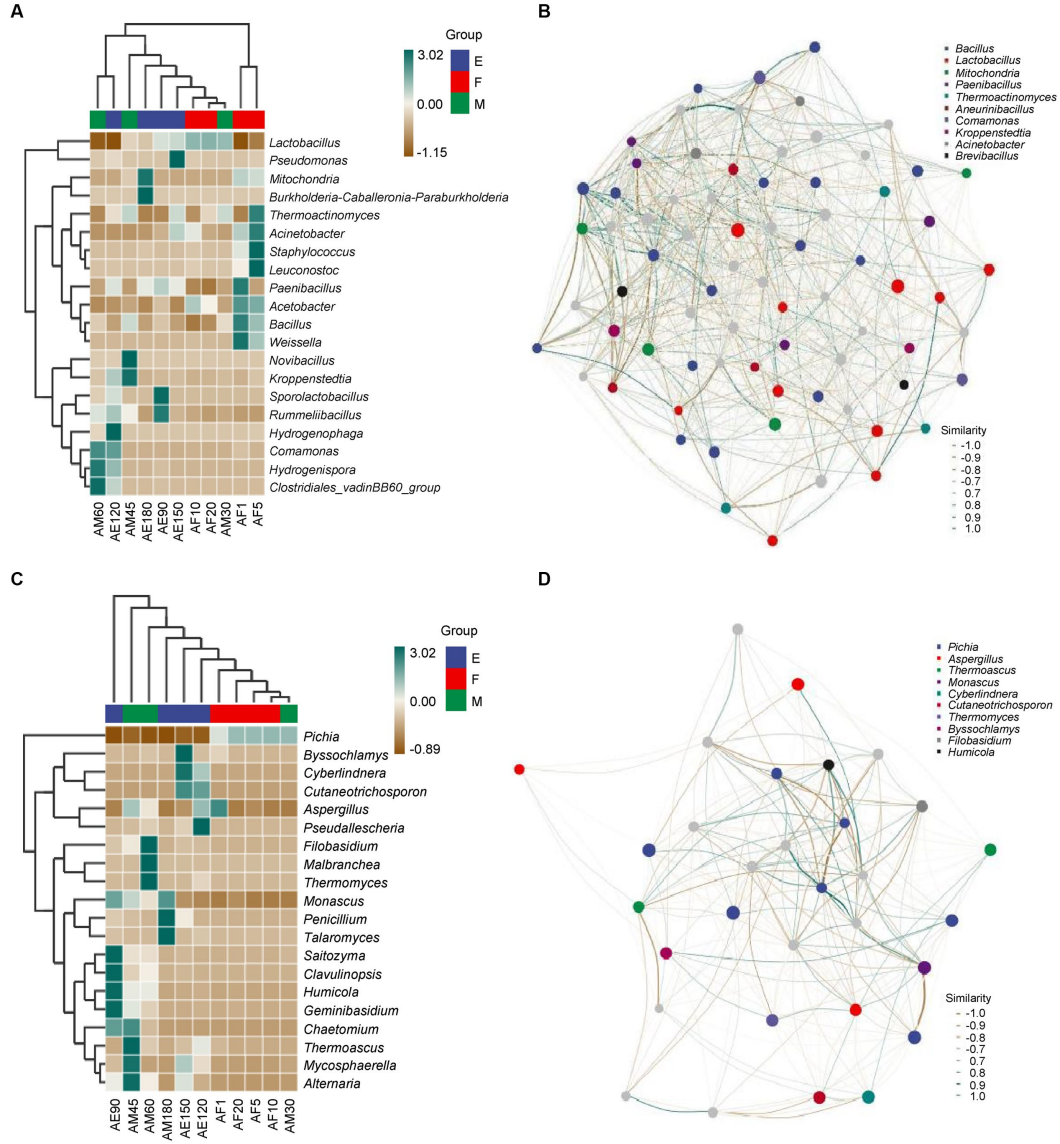
Heatmap and correlation network diagram of the microbial community in FG during the ultra-long fermentation process. **(A)** Heatmap of bacteria, **(B)** correlation network diagram of bacteria, **(C)** heatmap of fungal, and **(D)** correlation network diagram of fungal.

As shown in Figure 3A, there were eight, nine, two, and two species of bacteria with an abundance greater than 1.00% in the early stages of the ultra-long fermentation. There was no common dominance of bacterial genera, and there were at least two bacterial genera with high abundance (more than 1.00%) that are important bacterial genera in FG, such as *Lactobacillus* and *Bacillus*. The relative abundances of *Lactobacillus*, *Mitochondria*, and *Burkholderia* were 34.02, 48.95, and 8.04% at the end of the ultra-long fermentation. These were the dominant bacterial genera in FG. In addition, *Lactobacillus* were identified as the most abundant bacteria during the ultra-long fermentation of CFB, which was consistent with the results revealed in previous studies (Pang et al., 2018). However, the significant difference between this work and previous studies was the prokaryotic microbial community structure in FG at different stages during the ultra-long fermentation process.

According to Figure 3C, there were two, two, one, and one species of fungi with relative abundances greater than 1.00% during the early stages of the ultra-long fermentation. There was no common dominance of fungal genera in FG, and at least two of the fungal genera with high abundance (more than 1.00%) are considered important in FG, such as *Pichia* and *Monascus*. In our research, *Pichia*, *Monascus*, and *Aspergillus* were considered to be the main fungal genera during different stages of the ultra-long fermentation. During the early stage, the relative abundances of *Pichia* reached 99.54%. However, at the late stage, the relative abundances of *Pichia* reduced to 0.12%, which indicated that *Pichia* played vital roles in the early stage of fermentation. *Pichia* can play a key role in aroma production and metabolism. *Monascus* plays an important role in promoting the generation of esters, whereas *Aspergillus* can produce a variety of enzymes, organic acids, and fatty acids to facilitate the generation of aromatic esters (Wang et al., 2019; Xu et al., 2021, 2022; Cheng et al., 2023). The relative abundances of *Pichia* were 58.33, 97.07, 99.54, and 96.65% in the early stages of the ultra-long fermentation, which may have been influenced by the stacking fermentation process (Wang et al., 2019; Cheng et al., 2022). The relative abundances of *Monascus*, *Penicillium*, and *Talaromyces* at the end of the ultra-long fermentation (AE180) were 72.04, 16.91, and 9.99%, respectively. This indicated microbial communities were dominated by esterification microorganisms at the late stage of the ultra-long fermentation.

Microbial association networks are predominantly used to clarify the assembly differences of community species caused by environmental differences or experimental treatments, with the aim of finding key bacterial groups or species that can leverage changes in community composition (Tang et al., 2023). The composition and function of the core microbial flora determine the style and quality of Baijiu (Wang et al., 2020; Shen et al., 2021). As shown in Figure 3B, *Bacillus*, *Lactobacillus*, and *Mitochondria* were positively correlated with other bacterial genera. *Bacillus* and *Lactobacillus* had pronounced negative correlations. *Bacillus* positively correlated with *Comamonas* and *Mitochondria*. As shown in Figure 3D, *Pichia*, *Aspergillus*, and *Thermoascus* were positively correlated with other fungal genera. There was a pronounced negative correlation between *Pichia*, *Aspergillue*, and *Thermoascus*. *Monascus* was positively correlated with *Filobasidium* and *Aspergillus*. Amplicon analysis revealed fundamental information about microbial succession and the positive correlations between the main microbiota structure and major endogenous factors. Furthermore, existence of correlations between the main microflora with important flavor metabolites (Song et al., 2017; Wang et al., 2019). In addition, these six genera are play important roles in the process of the major flavor metabolites and formation in fermented grains, indicating that these six genera were the main microflora in FG during the ultra-long fermentation period.

*S. cerevisiae* plays a dominant role in the succession of fungal communities (Liu et al., 2021; Shen et al., 2021). Microorganisms are constantly domesticated during ultra-long fermentation, such as *Lactobacillus* and *Monascus*, among which *Monascus* plays an important role in promoting the formation of esters such as ethyl caproate and ethyl acetate (Xu et al., 2021, 2022). The influences of environmental factors and the interaction among microorganisms led to differences in fungal species and abundance in FG, including *Monascus*, *Penicillium*, and *Talaromyces*. These factors also influenced bacteria in FG, such as *Lactobacillus*, *Mitochondria*, and *Burkholderia*.

#### 3.2.4. Changes of volatile components in FG during ultra-long fermentation

**Figure 4:**
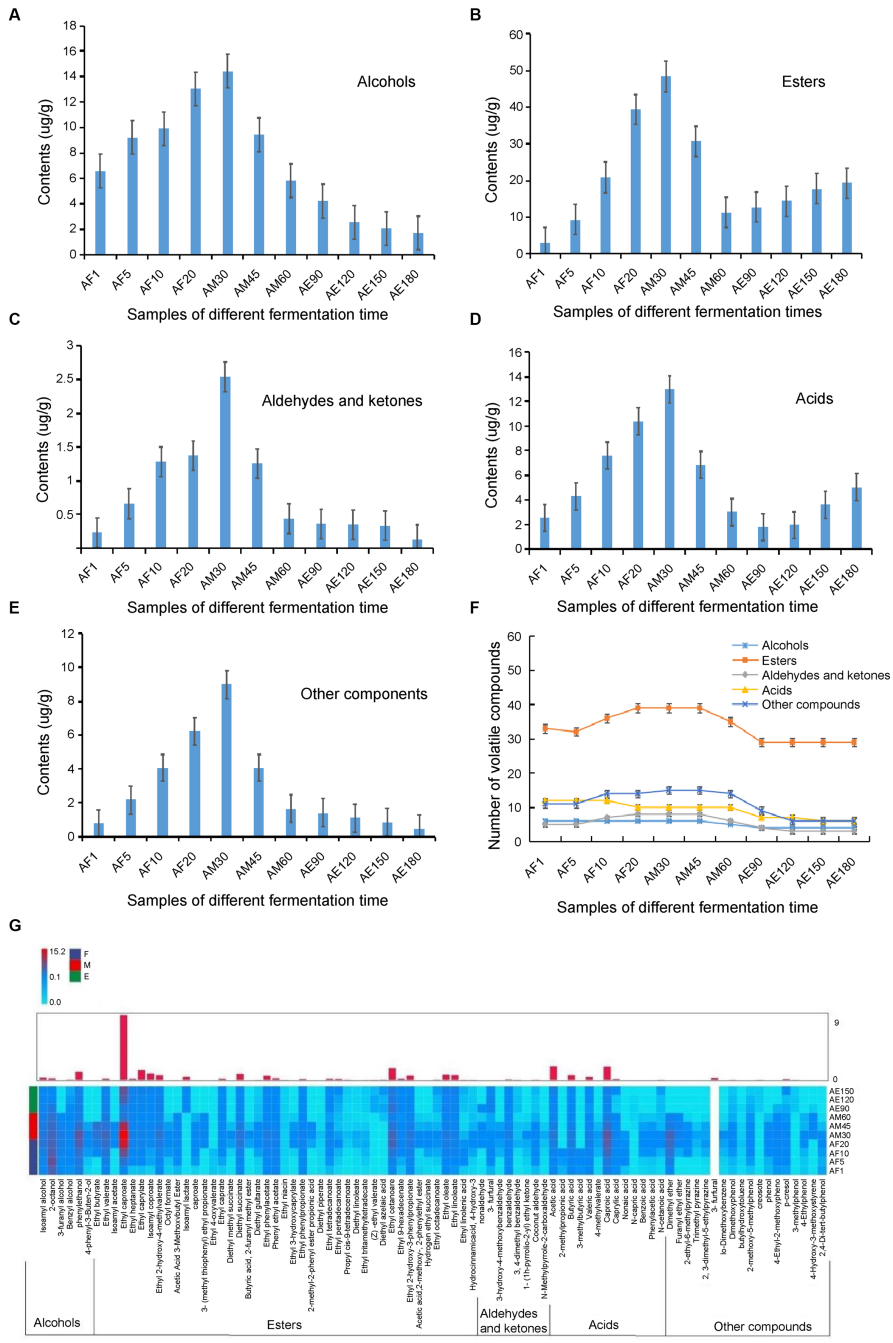
Categories and contents of volatile components in FG at different stages during the ultra-long fermentation process. **(A)** Alcohol, **(B)** asters, **(C)** aldehydes and ketones, **(D)** acid, **(E)** other classes, **(F)** number changes of volatile components, and **(G)** heat map of 88 metabolites in FG at different fermentation time.

Extraction and concentration processes were performed simultaneously using headspace solid-phase microextraction, which is a simple, rapid, and inexpensive technique. The required sample volumes are also relatively small (Wang et al., 2020). A total of 88 volatiles, including six alcohols, 43 esters, eight aldehydes and ketones, 13 acids, and 18 other compounds, were detected in FG using HS-SPME-GC–MS (Figure 4F and Supplementary Table S3). These volatile components were accurately characterized, and so many volatile components were detected may related to the time of ultra-long fermentation. Hu et al. (2021) reported that a total of 71 volatiles including 33 esters, 14 alcohols, 9 fatty acids, 5 phenols, and 10 other compounds were detected by HS-SPME-GC–MS in FG of strong-flavor Baijiu. As we know, the RI comparison is a method to identify unknown compounds by comparing the RI of an unknown compound and known compound. If their RI are similar, the unknown compound may be considered as the known compound (Chen et al., 2022). In our research, RI combination with MS as an auxiliary qualitative method (Supplementary Table S3), the results showed that most of the important aroma substances were formed in the early stages, and some, including ethyl caproate, acetic acid, and creosote, increased during the later stages of the ultra-long fermentation process (Figure 4G). The unique volatile content in FG increased sharply from days 1 to 30, and these compounds were mainly esters (Figures 4B,F).

The number of volatile compounds in FG in the later stage was lower than that in the early stage. The content of esters, alcohols, and others in the middle stage was the highest among the three different stages and was highest on the 30th day (Figure 4). Tang et al. (2022) identified 64 major volatile in fermented grains of light-flavor Xiaoqu baijiu by HS-SPME-GC–MS, which fermented for 98 days, and indicated that the contents of esters and alcohols increased, while a noteworthy decrease of the acids, aldehydes and ketones, and others contents was observed along with the fermentation time. In this study, the content of many volatiles assigned to esters, alcohols, and others showed significant differences during the ultra-long fermentation process for 180 days. Comparing with the normal fermentation (60 days), the content and proportion of esters and acids are increased during the ultra-long fermentation process (180 days). To our knowledge, the quality of baijiu is determined by the content and proportion of flavor compounds that distilled from FG, which are related to the variety and content of flavour compounds in FG. Herein, the appropriate fermentation time of FG is important to improve the quality of baijiu.

### 3.3. Correlation analysis of the microbial community with physicochemical parameters and volatile components

#### 3.3.1. RDA analysis of microbial community and physicochemical parameters

**Figure 5:**
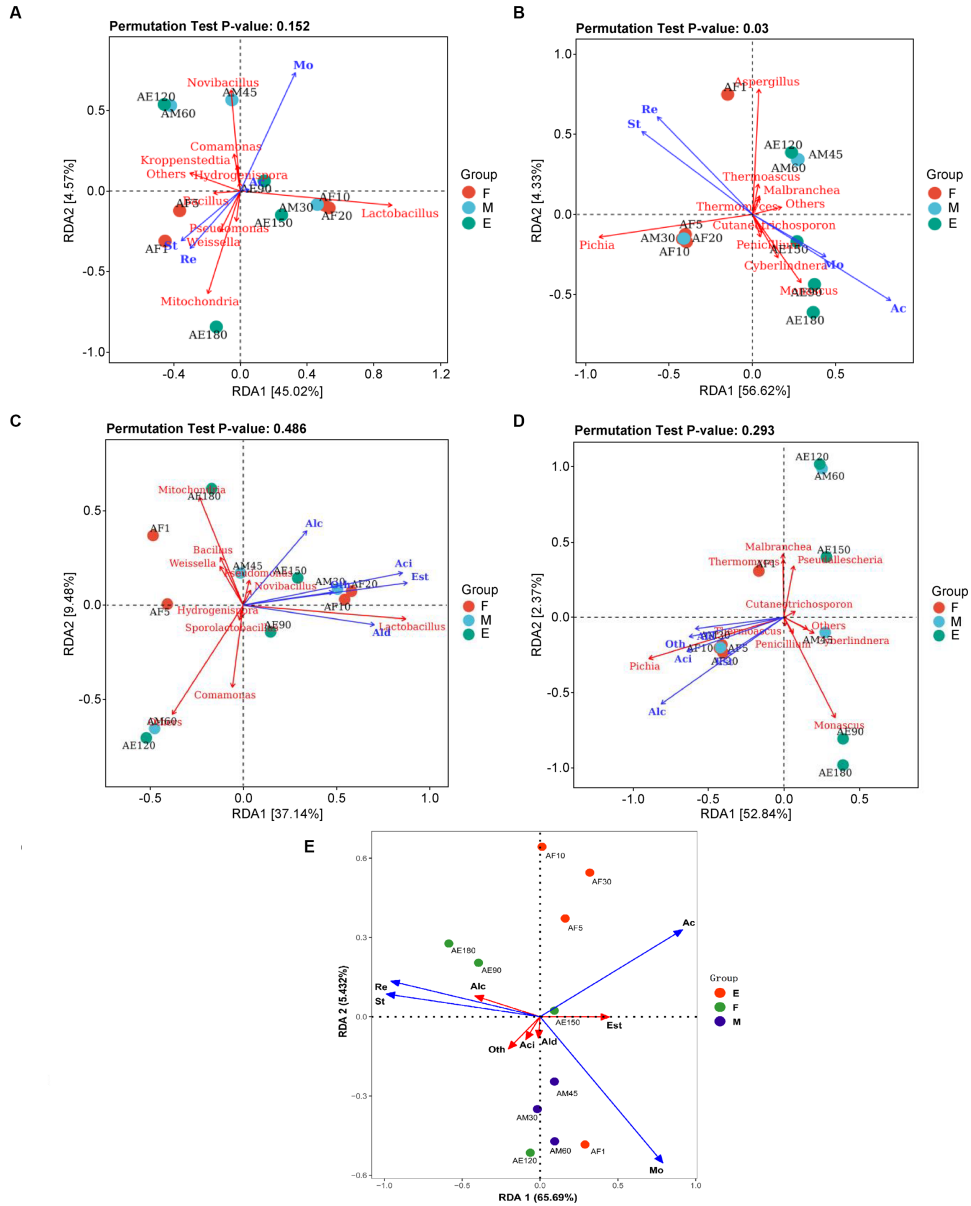
RDA analysis of dominant microorganisms at the genus level in FG during the ultra-long fermentation process. **(A)** RDA analysis of physicochemical indexes and bacterial genera, **(B)** RDA analysis of physicochemical indexes and fungi genera, **(C)** RDA analysis of volatile components and bacteria genus, **(D)** RDA analysis of volatile components and fungi genera, **(E)** RDA analysis of physicochemical indexes and volatile components. For **(A–E)**: Mo (moisture, %), Ac (acidity, n mol/10 g), St (starch, g/100 g), Re (reducing sugar, g/100 g), Alc (alcohols, %), Ald (aldehydes and ketones, %), Aci (acids, %), Est (esters, %), and Oth (other classes, %).

For bacteria (Figure 5A), *Bacillus*, *Pseudomonas*, *Weissella*, and *Mitochondria* were positively correlated with reducing sugar and starch, and negatively correlated with acidity and moisture. *Lactobacillus* was positively correlated with acidity and moisture, and negatively correlated with starch and reducing sugar content. Acidity had little effect on the composition and function of the bacterial flora. For fungi (Figure 5B), *Pichia* was positively correlated with reducing sugars and starch and negatively correlated with acidity and moisture. *Aspergillus* was positively correlated with reducing sugar and starch and negatively associated with acidity and moisture.

Reducing sugars, acidity, and temperature determine and maintain bacterial community changes during the fermentation process, and *S. cerevisiae* plays a dominant role in the succession of the fungal community (Liu et al., 2021). The *p*-values of the permutation test indicated that the effect of environmental factors on the composition and function of the fungal flora (pa = 0.152) was higher than that on the bacterial flora (pb = 0.003). It is well known that physicochemical properties (moisture content, acidity, residual starch, and sugar contents) are responsible for the microbial community changes that occur in microbial ecosystems (Jung et al., 2014). Luo et al. (2023) reported that total acidity and reducing sugar of FG played important roles in promoting the formation of core microbiota and succession of dominant taxa, which are similar to our research results. In addition, the more thorough fermented grains caused by the extended fermentation time resulted in the changes of these physicochemical indicators.

The physicochemical parameters of FG, such as starch and reducing sugars, are important factors affecting the composition and function of the bacterial and fungal flora. However, acidity had little effect on the composition and function of the bacterial flora. Previous studies demonstrated high concentrations of water, ethanol as well as high acidity could facilitate the growth of *Lactobacillus* in the brewing process (Liu and Miao, 2020). The high acid environment in the later stages of ultra-long fermentation formed a micro-environment that was conducive to the growth of *Lactobacillus*, which promoting the increase of acid in FG (Supplementary Figure S2A).

#### 3.3.2. RDA analysis of microbial community and volatile components

A total of 37.14 and 52.84% of the variation in metabolites was explained in groups by the top two RDA axes, indicating that the correlations between microbial communities and volatile components were strong (Figures 5C,D). This indicated that most of the volatile components, including alcohols, acids, and esters, were positively correlated with the dominant genera of FG, including *Pseudomonas, Lactobacillus, Novibacillus, Pichia, and Thermoascus*. *Lactobacillus* is regarded as the core functional microorganism responsible for increasing acidity, and can produce lactic acid, ethanol, and acetic acid through heterolactic fermentation (Song et al., 2017; Tang et al., 2019). *S. cerevisiae* can metabolize ethanol and provide acid resistance during the Baijiu brewing process. This plays an important role in the diversity of tastes and flavors with established qualities (Wang et al., 2019, 2020; Xu et al., 2022). *Pichia* is the main non-alcoholic yeast during the Baijiu brewing process and is predominantly used to form volatile compounds in Baijiu (Wang et al., 2021).

The production of volatile compounds, such as alcohols and esters, is related to the degradation of different sugars and amino acids by microorganisms, which can also lead to the generation of other volatile compounds such as furanones and pyrazines during fermentation (Lee et al., 2019). However, some microbes had the activity to coordinate with flavor-producing microbes to improve flavor compounds, but they are not flavor compound producers (Wang et al., 2019). The essence of baijiu-brewing is the process of microbial growth and accumulation of metabolites, and the synergistic effect among populations in FG is pivotal related to the flavor compounds and quality of baijiu. In addition, it reported that the environmental microbiota can drive microbial succession during fermentation, and flavor metabolism of microbial could subtly shaping the quality of baijiu (Luo et al., 2023). Meanwhile, our research results indicating that microbial succession influence by physicochemical parameters of FG, also. To our knowledge, CFB fermentation is a multifarious microecological fermentation system and open fermentation environment. Therefore, the changes in microbial succession and flavor metabolism during this brewing process require further study.

#### 3.3.3. RDA analysis of physicochemical parameters and volatile components

A total of 65.69 and 5.43% of the variation in metabolites was explained in groups by the top two RDA axes, indicating that the correlations between physicochemical parameters and volatile components were strong (Figure 5E). This indicated that most of the volatile components, including alcohols, acids, and esters, were positively correlated with physicochemical properties of FG. In our research, we found that the reducing sugar and starch of FG are positively correlated with alcohols, but negatively correlated with esters. In addition, the acidity of FG are positively correlated with esters. Base on the above dates, indicating that the changes of physicochemical parameters will influence the kinds or contents of volatile components in FG. Further, to improve the quality and aroma of baijiu by the control of physicochemical parameters of FG during fermentation process, is possible.

The microbial composition and environmental factors can take a great influence on community succession during fermentation of Baijiu (Tang et al., 2022), indicating that those two factors are the main driver that induced the succession of fermentation from the front to the end stage. To our knowledge, microbial community and physicochemical parameters of FG is interactional, which lead to the differences of microbial metabolisms including flavor compounds.

#### 3.3.4. Association network analysis on microbial community and volatile components

**Figure 6:**
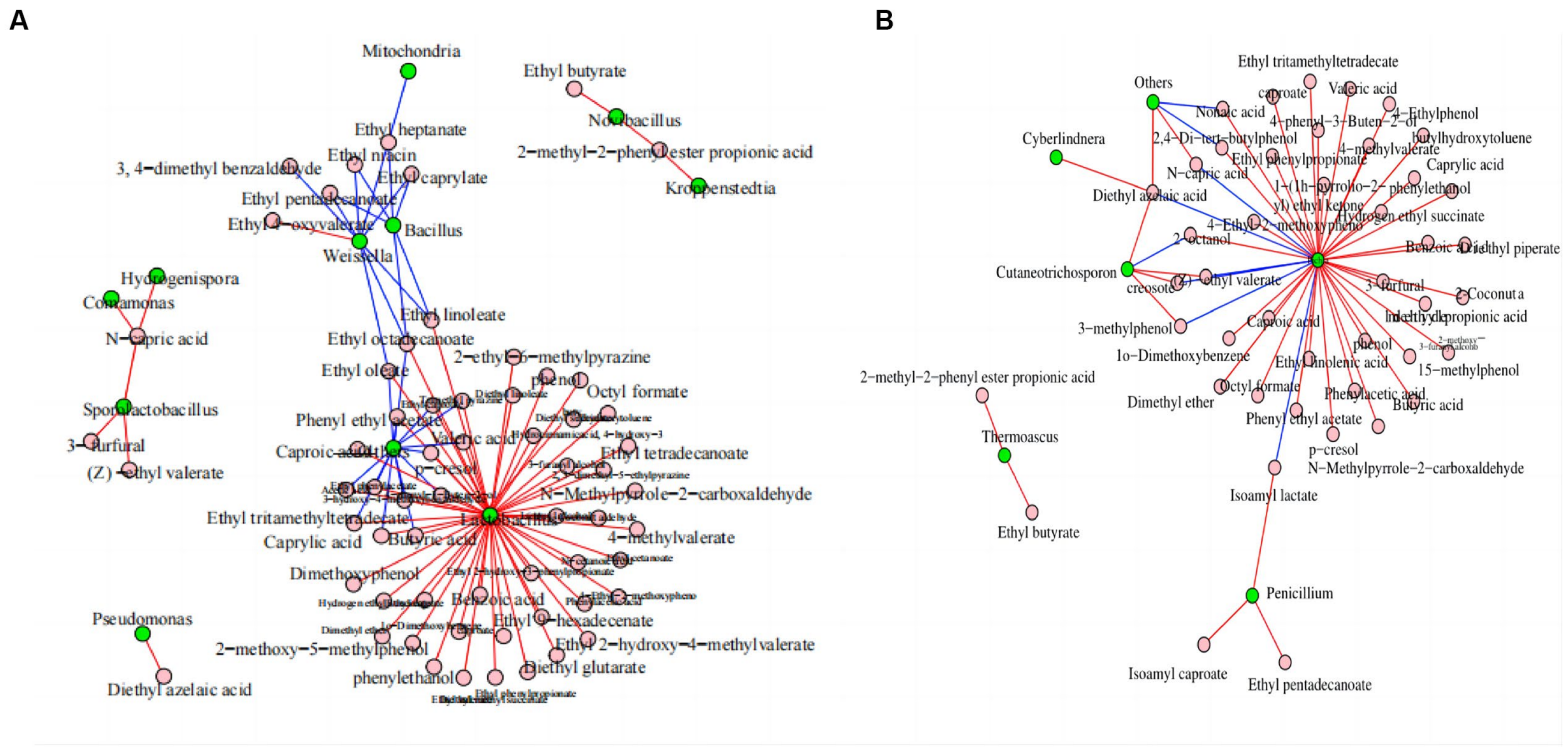
Correlation network diagram of dominant microorganisms at the genus level in FG during the ultra-long fermentation process. **(A)** Correlation network diagram of bacteria with volatile components and **(B)** correlation network diagram of fungal with volatile components. For **(A)** and **(B)**, the nodes represent the kings of connections between microorganisms or volatile components; the blue line represents a negative correlation, and the red line represents a positive correlation.

Network analysis was conducted to evaluate the effects of microorganisms on flavor substances. Figure 6A shows that *Lactobacillus* was the bacterial genus with the largest number of connections and that it was positively correlated with the anabolism of 48 volatile components, including ethyl caproate, butyric acid, and trimethyl pyrazine. *Bacillus* was negatively correlated with the anabolism of six volatile components. *Weissella* was negatively correlated with the anabolism of eight volatile components, including ethyl caproate, ethyl heptanate, and ethyl niacin. *Bacillus subtilis* and *Bacillus licheniensis* can metabolize and produce many flavor compounds that are essential to the quality of Maotai-flavor Baijiu (Li et al., 2014; Xu et al., 2022).

*Pichia* was the fungal genus with the largest number of connections, and it was positively correlated with the anabolism of 34 volatile components, including ethyl caproate, caproic acid, and 3-furfural. Moreover, it was negatively correlated with the anabolism of six volatile components, including isoamyl lactate, (Z)-ethyl valerate, and N-capric acid (Figure 6B). *Pichia* is an important aroma-producing fungus in the brewing of Baijiu, which can increase the enrichment of volatile flavors, such as acids and esters (Wang et al., 2021; Zhang et al., 2021; Huang et al., 2023).

In conclusion, *Lactobacillus, Bacillus, Weissella*, and *Pichia* were the core microbial genera involved in metabolizing the volatile components of FG. This provided abundant volatile components and precursors for CFB brewing and contributed to the style and aroma formation of CFB. Functional correlations between the core microbiota and important metabolites, such as volatile components, remain to be established and researched in the CFB brewing process (Song et al., 2017; Wang et al., 2019). In addition, considerable research is required to identify the correlations between microbial and volatile components in FG, especially to identify the functional microorganisms at species level, and we are planning to tackle these questions in future work to reveal the functional microorganisms in FG. In addition, we are playing to study on spatial heterogeneity of active microbial community in FG based on meta transcriptome and culture-dependent, and those works will be benefit for the explain and clear of the mechanism related to the changes of physicochemical parameters, microbial community and volatile components during ultra-long fermentation process of CFB.

## 4. Conclusion

Herein, the main microbes in FG were *Bacillus*, *Lactobacillus*, *Mitochondria*, *Pichia*, *Aspergillus*, and *Thermoascus* during ultra-long fermentation of CFB. Physicochemical parameters, such as starch and reducing sugars, were important factors affecting the composition and function of bacteria and fungi. In generally, organic acids of FG were produced by bacterial genera such as *Lactobacillus*, *Clostridium*, and *Acetobacter*. In addition, *Bacillus*, *Pichia*, *Wickerhamomyces*, and *Saccharomyces* are important genera affecting the volatile component content, including esters and alcohols. Herein, *Lactobacillus, Bacillus, Weissella*, and *Pichia* were the core microbial genera involved in the metabolism of volatile components in FG. These results help clarify the fermentation mechanisms and offer a theoretical reference to control and improve the quality of CFB.

In a word, ultra-long fermentation time changes the overall flavor balance of baijiu and improve the yielding of some esters, reduce the contents and numbers of other components, which may be conducive to the production of special flavoring baijiu (Luo et al., 2023). Herein, the clear relationships of physicochemical parameters, microbial community and volatile components in FG during fermentation, is important to reveal the fermentation mechanism of brewing and select the appropriate fermentation time to improve the aroma and yield of baijiu. Further research is necessary to identify core microbial genera that affect volatile components in FG, the response and succession mechanisms of these genera to the brewing environment, and their effects on the aroma and quality of CFB. Future research should search for the aroma characteristics of CFB, including the microbial community structure and its metabolites at different fermentation stages. The results expand our understanding on the relationships of physicochemical parameters, microbial community and volatile components of FG, and those factors that influenced the quality and aroma of CFB. Further, to improve the quality and aroma of baijiu by control of fermentation conditions, adjustment of fermentation time and microbial community.

## Data availability statement

The data presented in the study are deposited in the National Center for Biotechnology Information (NCBI) repository, accession number PRJNA1026830.

## Author contributions

WC: Writing – original draft. XuC: Writing – review & editing. WL: Writing – review & editing. GL: Writing – review & editing. XX: Data curation, Software, Writing – review & editing. RL: Writing – review & editing. TP: Data curation, Software, Writing – review & editing. NL: Data curation, Software, Writing – review & editing. DZ: Writing – review & editing. XiC: Data curation, Software, Writing – review & editing.
