# Supplementary material for: Insights into the influence of physicochemical parameters on the microbial community and volatile compounds during the ultra-long fermentation of compound-flavor Baijiu
Source: Front Microbiol. 2023 Oct 26;14:1272559. doi: 10.3389/fmicb.2023.1272559 (PMC10641013; doi:10.3389/fmicb.2023.1272559)
Supplement: Supplementary file 1 [file Table_1.DOCX]

**Table S1. Alpha diversity indices of bacterial metagenomes of fermented grains samples.**

| **Sample** | **Coverage** | **Shannon** | **Simpson** | **Ace** | **Chaol** |
| --- | --- | --- | --- | --- | --- |
| AF1 | 0.999913 | 5.10902 | 0.918168 | 366 | 367.408 |
| AF5 | 0.999911 | 5.64973 | 0.953541 | 470.2 | 472.04 |
| AF10 | 0.999866 | 1.77161 | 0.54282 | 258.1 | 261.375 |
| AF20 | 0.999954 | 1.82329 | 0.576492 | 150.7 | 151.291 |
| AM30 | 0.999964 | 2.21044 | 0.610744 | 174.5 | 175.228 |
| AM45 | 0.999986 | 3.64024 | 0.848621 | 165.8 | 165.842 |
| AM60 | 0.999965 | 5.76277 | 0.923102 | 533.7 | 534.075 |
| AE90 | 0.999979 | 3.90052 | 0.860182 | 252.8 | 253.065 |
| AE120 | 0.999971 | 5.07713 | 0.894241 | 423.8 | 424.145 |
| AE150 | 0.999995 | 2.39528 | 0.705084 | 166 | 166 |
| AE180 | 0.999989 | 2.69373 | 0.731852 | 219 | 219.125 |
