# Supplementary material for: Insights into the influence of physicochemical parameters on the microbial community and volatile compounds during the ultra-long fermentation of compound-flavor Baijiu
Source: Front Microbiol. 2023 Oct 26;14:1272559. doi: 10.3389/fmicb.2023.1272559 (PMC10641013; doi:10.3389/fmicb.2023.1272559)
Supplement: Supplementary file 2 [file Table_2.DOCX]

**Table S2. Alpha diversity indices of fungal metagenomes of fermented grains samples.**

| **Sample** | **Coverage** | **Shannon** | **Simpson** | **Ace** | **Chaol** |
| --- | --- | --- | --- | --- | --- |
| AF1 | 0.999989 | 2.22588 | 0.730888 | 63.7 | 63.7617 |
| AF5 | 0.999981 | 1.95602 | 0.682681 | 53.5 | 53.6783 |
| AF10 | 0.999964 | 2.06701 | 0.64658 | 47.7 | 48.4121 |
| AF20 | 0.999965 | 2.10237 | 0.713972 | 60.7 | 61.1882 |
| AM30 | 0.999986 | 2.03251 | 0.699835 | 60 | 60.1583 |
| AM45 | 0.999991 | 3.67764 | 0.820985 | 106 | 106.15 |
| AM60 | 0.999994 | 3.86202 | 0.85393 | 175 | 175.017 |
| AE90 | 0.999979 | 3.7379 | 0.600856 | 556.9 | 557.177 |
| AE120 | 0.999997 | 3.22443 | 0.832411 | 102 | 102.1 |
| AE150 | 0.999984 | 2.85808 | 0.722398 | 115 | 115.118 |
| AE180 | 0.999988 | 1.28635 | 0.451325 | 41 | 41.12 |
