# Supplementary material for: Insights into the influence of physicochemical parameters on the microbial community and volatile compounds during the ultra-long fermentation of compound-flavor Baijiu
Source: Front Microbiol. 2023 Oct 26;14:1272559. doi: 10.3389/fmicb.2023.1272559 (PMC10641013; doi:10.3389/fmicb.2023.1272559)
Supplement: Supplementary file 3 [file Table_3.DOC]

**Table S3. Categories and contents of volatile components in fermented grains during the ultra-long fermentation process.**

| No. | RI | Volatile components | Content（μg /g） | | | | | | | | | | |
| --- | --- | --- | --- | --- | --- | --- | --- | --- | --- | --- | --- | --- | --- |
|  |  |  | Early stage of ultra-long fermentation | | | | Middle stage of ultra-long fermentation | | | Late stage of ultra-long fermentation | | | |
|  |  |  | AF1 | AF5 | AF10 | AF20 | AM30 | AM45 | AM60 | AE90 | AE120 | AE150 | AE180 |
|  |  | **Alcohols** |  | | | | | | | | | | |
| 1 | 1110 | Isoamyl alcohol | 0.1421±0.0000 | 0.5767±0.0010 | 0.9139±0.0176 | 2.3783±0.0839 | 2.0234±0.0028 | 1.5805±0.0946 | 1.1816±0.0630 | 0.8942±0.0139 | 0.6254±0.0265 | 0.4839±0.0089 | 0.3080±0.0018 |
| 2 | 1327 | 2-octanol | 4.6503±0.4349 | 6.4316±0.0732 | 5.0255±0.0215 | 4.3826±0.0256. | 4.0679±0.0020 | 3.5236±0.0362 | 2.8675±0.0213 | 1.9964±0.2950 | 0.6436±0.0005 | 0.3580±0.0120 | 0.2137±0.0032 |
| 3 | 1555 | 3-furanyl alcohol | 0.2771±0.0004 | 0.3089±0.0007 | 0.8650±0.0005 | 1.1965±0.0039 | 1.7583±0.0068 | 0.8548±0.0472 | 0.2324±0.0122 | ND | ND | ND | ND |
| 4 | 1766 | Benzyl alcohol | 0.0908±0.0000 | 0.1096±0.0000 | 0.1253±0.0001 | 0.3664±0.0040 | 0.4797±0.0003 | 0.2563±0.0023 | 0.1201±0.0002 | 0.0986±0.0000 | 0.0806±0.0000 | 0.0639±0.0000 | 0.0492±0.0000 |
| 5 | 1802 | phenylethanol | 1.3953±0.0057 | 1.7750±0.0166 | 2.9199±0.0135 | 4.6464±0.4928 | 5.9439±0.0479 | 3.1336±0.4936 | 1.3910±0.5155 | 1.2553±0.0604 | 1.2044±0.0012 | 1.1880±0.0397 | 1.1402±0.0262 |
| 6 | 1958 | 4-phenyl-3-Buten-2-ol | 0.0153±0.0000 | 0.0325±0.0000 | 0.0782±0.0000 | 0.1023±0.0001 | 0.1314±0.0000 | 0.0685±0.0003 | ND | ND | ND | ND | ND |
|  |  | **Esters** |  | | | | | | | | | | |
| 1 | 851 | Ethyl butyrate | ND | ND | 0.2661±0.0000 | 1.4743±0.2819 | 2.4182±0.3272 | 2.8029±0.6252 | ND | ND | ND | ND | ND |
| 2 | 1039 | Ethyl valerate | 0.4201±0.0023 | 1.2500±0.0019 | 2.2200±0.0055 | 3.0235±0.0024 | 4.1097±0.0049 | 2.4823±0.0002 | 1.6801±0.0052 | 1.1203±0.0036 | 0.8678±0.0284 | 0.8511±0.0000 | 0.1781±0.0041 |
| 3 | 1069 | Isoamyl acetate | ND | ND | ND | 0.1095±0.0005 | 0.2093±0.0004 | 0.1794±0.0027 | ND | ND | ND | ND | ND |
| 4 | 1100 | Ethyl caproate | 0.3118±0.0006 | 2.6396±0.0261 | 6.9142±0.0334 | 12.8720±1.9459 | 15.2077±2.6124 | 10.9176±0.8623 | 2.3910±0.0924 | 1.7825±0.0842 | 4.8688±0.0682 | 6.3152±0.1305 | 9.1623±0.3352 |
| 5 | 1238 | Ethyl heptanate | 0.0311±0.0000 | 0.1960±0.0000 | 0.4599±0.0002 | 0.8411±0.0059 | 1.0304±0.0057 | 0.6282±0.0175 | 0.6532±0.0047 | 0.6967±0.0002 | 0.7498±0.0176 | 0.8868±0.0824 | 0.1635±0.0002 |
| 6 | 1341 | Ethyl caprylate | 0.0388±0.0000 | 0.1760±0.0028 | 0.5294±0.0011 | 0.9012±0.0067 | 1.1043±0.0275 | 0.7053±0.0193 | 0.1898±0.0033 | 0.5515±0.0195 | 0.7043±0.0448 | 1.0906±0.0398 | 1.3916±0.0156 |
| 7 | 1361 | Isoamyl caproate | 0.0213±0.0000 | 0.0684±0.0012 | 0.0725±0.0000 | 0.4627±0.0015 | 0.5807±0.0043 | 0.3722±0.0086 | 0.3514±0.0081 | 0.3069±0.0001 | 0.2641±0.0148 | 0.4779±0.0032 | 0.8989±0.0145 |
| 8 | 1440 | Ethyl 2-hydroxy-4-methylvalerate | 0.1273±0.0002 | 0.3350±0.0006 | 0.8765±0.0008 | 1.7837±0.0461 | 2.7163±0.0166 | 1.4675±0.1285 | 0.7921±0.0027 | 0.9511±0.0397 | 0.9371±0.0051 | 0.7685±0.0029 | 0.6710±0.0014 |
| 9 | 1450 | Octyl formate | 0.0214±0.0021 | 0.0349±0.0000 | 0.0877±0.0002 | 0.1198±0.0002 | 0.1126±0.0001 | 0.0778±0.0006 | 0.0205±0.0000 | ND | ND | ND | ND |
| 10 | 1466 | Acetic Acid 3-Methoxybutyl Ester | 0.0457±0.0000 | 0.0961±0.0000 | 0.2886±0.0009 | 0.5337±0.0040 | 0.8186±0.0016 | 0.4370±0.0113 | 0.4585±0.0080 | ND | ND | ND | ND |
| 11 | 1495 | Isoamyl lactate | ND | ND | ND | ND | ND | ND | ND | 0.2235±0.0009 | 0.2418±0.0005 | 0.3337±0.00000 | 0.4385±0.0029 |
| 12 | 1503 | caproate | 0.0166±0.0000 | 0.0811±0.0000 | 0.1495±0.0000 | 0.2662±0.0008 | 0.2231±0.0009 | 0.1096±0.0008 | 0.0583±0.0004 | ND | ND | ND | ND |
| 13 | 1507 | 3- (methyl thiophenyl) ethyl propionate | ND | ND | ND | 0.3352±0.0036 | 0.5813±0.0022 | 0.3110±0.0027 | 0.1161±0.0017 | ND | ND | ND | ND |
| 14 | 1509 | Ethyl 4-oxyvalerate | 0.0298±0.0000 | 0.0315±0.0000 | 0.0401±0.0006 | ND | ND | ND | ND | ND | ND | ND | ND |
| 15 | 1530 | Ethyl caprate | 0.0245±0.0002 | 0.0671±0.0008 | 0.1983±0.0006 | 0.3866±0.0033 | 0.5022±0.0012 | 0.2534±0.0032 | 0.2454±0.0000 | 0.2349±0.0029 | 0.1120±0.0004 | 0.1946±0.0038 | 0.1677±0.0010 |
| 16 | 1559 | Diethyl methyl succinate | ND | ND | 0.0171±0.0000 | 0.0754±0.0006 | 0.1290±0.0003 | 0.0423±0.0001 | ND | ND | ND | ND | ND |
| 17 | 1569 | Diethyl succinate | 0.2466±0.0015 | 0.4946±0.0015 | 0.9370±0.0014 | 2.3926±0.0001 | 2.5232±0.0023 | 1.6703±0.1504 | 0.6299±0.0087 | 0.4837±0.0023 | 0.4530±0.0009 | 0.5081±0.0008 | 0.8397±0.0104 |
| 18 | 1605 | Butyric acid, 2-furanyl methyl ester | ND | ND | ND | 0.0720±0.0000 | 0.1035±0.0000 | 0.0395±0.0000 | ND | ND | ND | ND | ND |
| 19 | 1667 | Diethyl glutarate | 0.0175±0.0000 | 0.0236±0.0000 | 0.0713±0.0000 | 0.1588±0.0001 | 0.2361±0.0004 | 0.1181±0.0013 | 0.0421±0.0004 | 0.0390±0.0000 | 0.0305±0.0000 | 0.0256±0.0000 | 0.0204±0000 |
| 20 | 1679 | Ethyl phenylacetate | 0.1135±0.0000 | 0.3007±0.0002 | 0.7244±0.0000 | 1.2010±0.0001 | 1.7413±0.0014 | 0.8328±0.0026 | 0.2810±0.0185 | 0.1928±0.0000 | 0.3480±0.0009 | 0.4397±0.0034 | 0.5925±0.0004 |
| 21 | 1710 | Phenyl ethyl acetate | 0.4614±0.0005 | 0.6482±0.0001 | 0.7833±0.0001 | 0.9603±0.0005 | 1.1909±0.0015 | 0.5893±0.0044 | 0.2212±0.0017 | 0.1529±0.0000 | 0.1275±0.0001 | 0.3507±0.0026 | 0.2221±0.0007 |
| 22 | 1714 | Ethyl niacin | 0.0103±0.0000 | 0.0145±0.0000 | 0.0190±0.0000 | 0.0207±0.0000 | 0.0234±0.0000 | 0.0259±0.0000 | 0.0223±0.0000 | 0.0192±0.0000 | 0.0181±0.0000 | 0.0204±0.0000 | 0.0224±0.0000 |
| 23 | 1798 | Ethyl 3-hydroxycaprylate | ND | ND | 0.0183±0.0000 | 0.1334±0.0008 | 0.1723±0.0002 | 0.0859±0.0001 | 0.0241±0.0001 | ND | ND | ND | ND |
| 24 | 1802 | Ethyl phenylpropionate | 0.1312±0.0000 | 0.2600±0.0010 | 0.4449±0.0000 | 0.8520±0.0200 | 1.0616±0.0058 | 0.5052±0.0000 | 0.1880±0.0074 | 0.1446±0.0000 | 0.1844±0.0002 | 0.3180±0.0029 | 0.1208±0.0002 |
| 25 | 1849 | 2-methyl-2-phenyl ester propionic acid | ND | ND | ND | 0.0649±0.0004 | 0.0966±0.0004 | 0.343±0.0000 | ND | ND | ND | ND | ND |
| 26 | 1906 | Diethyl piperate | 0.0092±0.0000 | 0.0124±0.0000 | 0.0170±0.0000 | 0.0235±0.0000 | 0.0378±0.0000 | 0.0121±0.0000 | 0.0066±0.0000 | ND | ND | ND | ND |
| 27 | 1919 | Ethyl tetradecanoate | 0.0446±0.0000 | 0.0862±0.0000 | 0.1534±0.0000 | 0.7361±0.0000 | 0.4885±0.0000 | 0.2403±0.0042 | 0.0763±0.0008 | 0.2040±0.0014 | 0.2101±0.0003 | 0.2875±0.0050 | 0.1646±0.0011 |
| 28 | 1970 | Ethyl pentadecanoate | 0.0089±0.0000 | 0.0247±0.0001 | 0.0502±0.0000 | 0.1038±0.0073 | 0.0772±0.0001 | 0.0372±0.0001 | 0.0432±0.0005 | 0.0562±0.0000 | 0.0709±0.0000 | 0.0995±0.0000 | 0.1395±0.0000 |
| 29 | 1973 | Propyl cis-9-tetradecenoate | ND | ND | 0.0045±0.0000 | 0.0260±0.0000 | 0.0442±0.0001 | 0.0185±0.0001 | 0.0163±0.0000 | 0.0122±0.0000 | 0.0189±0.0000 | 0.0257±0.0000 | 0.0376±0.0000 |
| 30 | 1979 | Diethyl linoleate | 0.0067±0.0000 | 0.0084±0.0000 | 0.0132±0.0000 | 0.0552±0.0000 | 0.0643±0.0000 | 0.0231±0.0000 | 0.0117±0.0000 | 0.0102±0.0000 | 0.0124±0.0000 | 0.0150±0.0000 | 0.0081±0.0000 |
| 31 | 1985 | Ethyl tritamethyltetradecate | 0.0098±0.0000 | 0.0262±0.0000 | 0.0425±0.0000 | 0.0603±0.0001 | 0.0535±0.0000 | 0.0266±0.0000 | 0.0064±0.0000 | 0.0216±0.0000 | 0.0247±0.0000 | 0.0291±0.0000 | 0.0174±0.0000 |
| 32 | 2060 | (Z) -ethyl valerate | ND | ND | ND | ND | ND | ND | ND | 0.0636±0.0000 | 0.0656±0.0005 | 0.0456±0.0005 | 0.0270±0.0001 |
| 33 | 2110 | Diethyl azelaic acid | ND | ND | ND | ND | ND | ND | 0.0336±0.0001 | 0.0675±0.0000 | 0.0500±0.0000 | 0.0859±0.0005 | 0.0424±0.0000 |
| 34 | 2117 | Ethyl cetanoate | 0.3438±0.0613 | 1.0562±0.0271 | 2.4102±0.0485 | 4.2886±0.6566 | 4.2080±0.2608 | 2.2723±0.3914 | 1.0805±0.0065 | 2.9007±0.7804 | 1.8814±0.0551 | 1.7577±0.3002 | 1.6646±0.0222 |
| 35 | 2142 | Ethyl 9-hexadecenate | 0.0281±0.0000 | 0.0584±0.0003 | 0.1229±0.0013 | 0.3843±0.0220 | 0.4350±0.0101 | 0.2055±0.0000 | 0.0912±0.0015 | 0.2443±0.0153 | 0.1724±0.0010 | 0.2797±0.0039 | 0.1705±0.0012 |
| 36 | 2155 | Ethyl 2-hydroxy-3-phenylpropionate | 0.3407±0.0005 | 0.5655±0.0005 | 0.9197±0.0001 | 1.6741±0.1186 | 2.1887±0.0083 | 1.1201±0.0572 | 0.5620±0.0021 | 0.4809±0.0076 | 0.4017±0.0000 | 0.2422±0.0007 | 0.6166±0.0036 |
| 37 | 2196 | Acetic acid,2-methoxy-, 2-phenylethyl ester | 0.0231±0.0000 | 0.0171±0.0000 | 0.0139±0.0000 | 0.124±0.0000 | 0.1110±0.0002 | 0.0564±0.0002 | 0.0260±0.0002 | ND | ND | ND | ND |
| 38 | 2242 | Hydrogen ethyl succinate | 0.0094±0.0000 | 0.0165±0.0000 | 0.0687±0.0000 | 0.1274±0.0021 | 0.2321±0.0043 | 0.0625±0.0006 | 0.0165±0.0001 | ND | ND | ND | ND |
| 39 | 2311 | Ethyl octadecanoate | 0.0065±0.0000 | 0.0247±0.0000 | 0.0610±0.0023 | 0.0933±0.0019 | 0.1078±0.0001 | 0.0529±0.0002 | 0.0244±0.0001 | 0.0529±0.0005 | 0.0513±0.0000 | 0.0788±0.0000 | 0.0445±0.0000 |
| 40 | 2332 | Ethyl oleate | 0.1321±0.0000 | 0.3034±0.0089 | 0.8260±0.0588 | 1.5220±0.4223 | 1.9448±0.1364 | 1.0132±0.0657 | 0.4848±0.0065 | 0.8531±0.1952 | 0.7502±0.0217 | 1.1470±0.0592 | 0.7419±0.0074 |
| 41 | 2381 | Ethyl linoleate | 0.0864±0.0001 | 0.1765±0.0031 | 0.9036±0.0365 | 0.9598±0.1313 | 1.3864±0.1815 | 0.5789±0.0051 | 0.3780±0.0072 | 0.8927±0.2492 | 0.7402±0.0435 | 1.0462±0.0592 | 0.6887±0.0232 |
| 42 | 2448 | Ethyl linolenic acid | 0.0241±0.0000 | 0.1765±0.0031 | 0.1331±0.0008 | 0.0909±0.0004 | 0.0489±0.0000 | 0.0134±0.0000 | 0.0098±0.0000 | 0.0363±0.0002 | 0.0381±0.0000 | 0.0549±0.0001 | 0.0298±0.0000 |
| 43 | 2707 | Hydrocinnamicacid, 4-hydroxy-3 | 0.0125±0.0000 | 0.0230±0.0000 | 0.0651±0.0001 | 0.1103±0.0012 | 0.1266±0.0002 | 0.0520±0.0003 | 0.0375±0.0005 | 0.0321±0.0002 | 0.0301±0.0000 | 0.0280±0.0000 | 0.0257±0.0000 |
|  |  | **Aldehydes and ketones** |  | | | | | | | | | | |
| 1 | 1337 | nonaldehyde | ND | ND | ND | 0.1385±0.0017 | 0.1998±0.0008 | 0.1015±0.0000 | 0.0265±0.0000 | 0.1133±0.0001 | ND | ND | ND |
| 2 | 1375 | 3- furfural | 0.0533±0.0000 | 0.3303±0.0004 | 0.6129±0.0049 | 0.1564±0.0004 | 0.2201±0.0005 | 0.0749±0.0000 | 0.0677±0.0019 | ND | ND | ND | ND |
| 3 | 1417 | 3-hydroxy-4-methoxybenzaldehyde | ND | ND | 0.0530±0.0000 | 0.0789±0.0000 | 0.1270±0.0017 | 0.0740±0.0008 | ND | ND | ND | ND | ND |
| 4 | 1435 | benzaldehyde | 0.1276±0.0002 | 0.2120±0.0003 | 0.3986±0.0001 | 0.5583±0.0074 | 1.3482±0.0941 | 0.7299±0.0541 | 0.2686±0.0117 | 0.1733±0.0083 | 0.2773±0.0019 | 0.2658±0.0005 | 0.0719±0.0000 |
| 5 | 1743 | 3, 4-dimethyl benzaldehyde | ND | ND | 0.0087±0.0000 | 0.0549±0.0001 | 0.0374±0.0009 | 0.0385±0.0000 | 0.0098±0.0000 | 0.0407±0.0000 | 0.0369±0.0000 | 0.0311±0.0000 | 0.0272±0.0000 |
| 6 | 1860 | 1- (1h-pyrrolio-2-yl) ethyl ketone | 0.0097±0.0000 | 0.0212±0.0000 | 0.0419±0.0000 | 0.0630±0.0002 | 0.1438±0.0005 | 0.0368±0.0000 | 0.0122±0.0000 | ND | ND | ND | ND |
| 7 | 1921 | Coconut aldehyde | 0.0404±0.0000 | 0.0947±0.0012 | 0.1486±0.0021 | 0.2954±0.0027 | 0.4028±0.0000 | 0.1833±0.0026 | 0.0587±0.0003 | 0.0321±0.0000 | 0.0321±0.0000 | 0.0448±0.0000 | 0.0356±0.0000 |
| 8 | 1990 | N-Methylpyrrole-2-carboxaldehyde | 0.0041±0.0000 | 0.0098±0.0000 | 0.0157±0.0000 | 0.0338±0.0000 | 0.0632±0.0008 | 0.0240±0.0000 | ND | ND | ND | ND | ND |
|  |  | **Acids** |  | | | | | | | | | | |
| 1 | 1352 | Acetic acid | 0.3616±0.0007 | 0.6235±0.0005 | 1.0065±0.0000 | 1.5665±0.0331 | 2.5205±0.1393 | 1.4419±0.2896 | 0.5942±0.0075 | 0.7695±0.0760 | 0.5634±0.0006 | 1.3999±0.0447 | 1.9110±0.0158 |
| 2 | 1462 | 2-methylpropionic acid | 0.0514±0.0000 | 0.0816±0.0000 | 0.1130±0.0001 | ND | ND | ND | ND | ND | ND | ND | ND |
| 3 | 1518 | Butyric acid | 0.3647±0.0008 | 0.5508±0.0016 | 0.9701±0.0024 | 1.5835±0.0332 | 1.9362±0.0074 | 1.0121±0.0645 | 0.3729±0.0308 | 0.1868±0.0021 | 0.2720±0.0004 | 0.3252±0.0000 | 0.6783±0.0000 |
| 4 | 1558 | 3-methylbutyric acid | 0.0881±0.0011 | 0.1551±0.0002 | 0.4558±0.0035 | ND | ND | ND | ND | ND | ND | ND | ND |
| 5 | 1623 | Valeric acid | 0.2878±0.0002 | 0.3719±0.0005 | 0.7359±0.0006 | 1.1089±0.0178 | 1.3119±0.0030 | 0.6855±0.0258 | 0.3055±0.0241 | 0.0858±0.0004 | 0.1807±0.0001 | 0.2279±0.0027 | 0.4125±0.0025 |
| 6 | 1686 | 4-methylvalerate | 0.0085±0.0000 | 0.0103±0.0000 | 0.0483±0.0000 | 0.0679±0.0001 | 0.0805±0.0000 | 0.0452±0.0001 | 0.0119±0.0000 | ND | ND | ND | ND |
| 7 | 1728 | Caproic acid | 1.2373±0.0051 | 2.0841±0.0135 | 3.6839±0.0132 | 5.0776±0.4632 | 5.9561±0.0595 | 3.1652±0.4729 | 1.4939±0.5463 | 0.6720±0.0263 | 0.8594±0.0035 | 1.4744±0.0412 | 1.8684±0.0037 |
| 8 | 1930 | Caprylic acid | 0.0958±0.0000 | 0.1967±0.0036 | 0.2551±0.0000 | 0.4532±0.0062 | 0.5527±0.0005 | 0.2771±0.0051 | 0.1044±0.0022 | 0.0232±0.0000 | 0.0285±0.0000 | 0.1063±0.0007 | 0.1253±0.0000 |
| 9 | 2031 | Nonaic acid | 0.0311±0.0001 | 0.1309±0.0185 | 0.1018±0.0001 | 0.0961±0.0019 | 0.0886±0.0094 | ND | ND | ND | ND | ND | ND |
| 10 | 2182 | N-capric acid | ND | ND | ND | ND | ND | 0.0091±0.0000 | 0.0164±0.0000 | 0.0183±0.0000 | 0.0127±0.0000 | ND | ND |
| 11 | 2304 | Benzoic acid | 0.0156±0.0000 | 0.0262±0.0000 | 0.0896±0.0001 | 0.1408±0.0003 | 0.1644±0.0002 | 0.0734±0.0003 | 0.0345±0.0001 | ND | ND | ND | ND |
| 12 | 2425 | Phenylacetic acid | 0.0217±0.0000 | 0.0333±0.0000 | 0.0914±0.0000 | 0.1708±0.0021 | 0.2339±0.0005 | 0.0821±0.0009 | 0.0309±0.0002 | ND | ND | ND | ND |
| 13 | / | N-cetanoic acid | 0.0112±0.0000 | 0.0140±0.0000 | 0.0499±0.0033 | 0.1155±0.0026 | 0.1383±0.0015 | 0.0472±0.0000 | 0.0451±0.0000 | 0.0421±0.0001 | 0.0411±0.0000 | 0.0781±0.0000 | 0.0298±0.0001 |
|  |  | **Other compounds** |  | | | | | | | | | | |
| 1 | / | Dimethyl ether | 0.3763±0.0078 | 1.5832±0.0288 | 2.7786±0.0282 | 3.7752±0.0098 | 4.5151±0.4974 | 1.6879±0.0879 | 0.8267±0.0027 | ND | ND | ND | ND |
| 2 | 1215 | Furanyl ethyl ether | ND | ND | 0.0594±0.0002 | 0.2572±0.0049 | 0.9930±0.0020 | 0.7302±0.0318 | 0.1994±0.0095 | ND | ND | ND | ND |
| 3 | 1315 | 2-ethyl-6-methylpyrazine | ND | ND | 0.0962±0.0001 | 0.1223±0.0001 | 0.2295±0.0005 | 0.0926±0.0005 | 0.0140±0.0000 | ND | ND | ND | ND |
| 4 | 1335 | Trimethyl pyrazine | ND | ND | 0.0258±0.0000 | 0.0492±0.0000 | 0.0720±0.0000 | 0.0314±0.0000 | ND | ND | ND | ND | ND |
| 5 | 1368 | 2, 3-dimethyl-5-ethylpyrazine | 0.0027±0.0000 | 0.0114±0.0000 | 0.0577±0.0001 | 0.0845±0.0000 | 0.0725±0.0000 | 0.0686±0.0004 | 0.0117±0.0002 | ND | ND | ND | ND |
| 6 | 1403 | 3- furfural | ND | ND | ND | ND | ND | ND | ND | 1.0931±0.0014 | 0.9152±0.0117 | 0.6253±0.0008 | 0.2550±0.0010 |
| 7 | 1620 | 1o-Dimethoxybenzene | 0.0116±0.0000 | 0.0208±0.0000 | 0.0480±0.0000 | 0.0702±0.0000 | 0.1419±0.0010 | 0.0338±0.0001 | 0.0115±0.0000 | ND | ND | ND | ND |
| 8 | 1752 | Dimethoxyphenol | 0.0341±0.0000 | 0.0492±0.0000 | 0.0860±0.0000 | 0.1993±0.0016 | 0.2742±0.0000 | 0.1089±0.0002 | 0.0579±0.0003 | 0.0484±0.0000 | 0.0413±0.0000 | 0.0313±0.0000 | 0.0305±0.0000 |
| 9 | 1798 | butylhydroxytoluene | 0.0075±0.0000 | 0.0158±0.0000 | 0.0219±0.0000 | 0.0347±0.0000 | 0.0240±0.0000 | 0.0207±0.0000 | 0.0086±0.0000 | 0.0148±0.0000 | ND | ND | ND |
| 10 | 1844 | 2-methoxy-5-methylphenol | 0.0306±0.0000 | 0.0811±0.0000 | 0.1260±0.0000 | 0.2753 ±.0026 | 0.3493±0.0007 | 0.1659±0.0016 | 0.0856±0.0019 | 0.0632±0.0000 | 0.0469±0.0000 | 0.0267±0.0000 | ND |
| 11 | 1880 | creosote | ND | ND | ND | ND | ND | ND | ND | 0.0245±0.0000 | 0.0258±0.0000 | 0.0272±0.0000 | 0.0286±0.0000 |
| 12 | 1888 | phenol | 0.0467±0.0000 | 0.0537±0.0000 | 0.1205±0.0000 | 0.1575±0.0005 | 0.1955±0.0000 | 0.0982±0.0004 | 0.0429±0.0006 | ND | ND | ND | ND |
| 13 | 1914 | 4-Ethyl-2-methoxypheno | 0.0803±0.0000 | 0.1485±0.0000 | 0.2595±0.0000 | 0.5309±0.0072 | 0.6730±0.0007 | 0.3156±0.0053 | 0.1206±0.0022 | 0.0529±0.0000 | ND | ND | ND |
| 14 | 1961 | p-cresol | 0.0766±0.0000 | 0.1333±0.0000 | 0.2588±0.0000 | 0.4668±0.0054 | 0.5646±0.0003 | 0.2950±0.0048 | 0.0862±0.0015 | 0.0632±0.0012 | 0.0401±0.0000 | 0.1083±0.0001 | 0.1109±0.0002 |
| 15 | 1997 | 3-methylphenol | ND | ND | ND | ND | ND | ND | ND | 0.0303±0.0000 | 0.0292±0.0000 | 0.0262±0.0000 | 0.0239±0.0000 |
| 16 | 2050 | 4-Ethylphenol | 0.0280±0.0000 | 0.0284±0.0000 | 0.0502±0.0000 | 0.0961±0.0008 | 0.2213±0.0761 | 0.0519±0.0000 | 0.0524±0.0019 | ND | ND | ND | ND |
| 17 | 2127 | 4-Hydroxy-3-methoxystyrene | ND | ND | ND | ND | 0.5704±0.0065 | 0.3261±0.0084 | 0.1265±0.0000 | 0.0243±0.0000 | ND | ND | ND |
| 18 | 2170 | 2,4-Di-tert-butylphenol | 0.0723±0.0004 | 0.0572±0.0006 | 0.0813±0.0000 | 0.1047±0.0039 | 0.0943±0.0001 | 0.0395±0.0000 | 0.0065±0.0000 | ND | ND | ND | ND |

ND, not detected. MS, compounds were identified by MS spectra. RI, compounds were identified by comparison with a pure standard. All the results are the average and error of triplicate experiments.
