# Supplementary material for: Insights into the influence of physicochemical parameters on the microbial community and volatile compounds during the ultra-long fermentation of compound-flavor Baijiu
Source: Front Microbiol. 2023 Oct 26;14:1272559. doi: 10.3389/fmicb.2023.1272559 (PMC10641013; doi:10.3389/fmicb.2023.1272559)
Supplement: Supplementary file 4 [file Data_Sheet_1.PDF]

Supplementary Figures

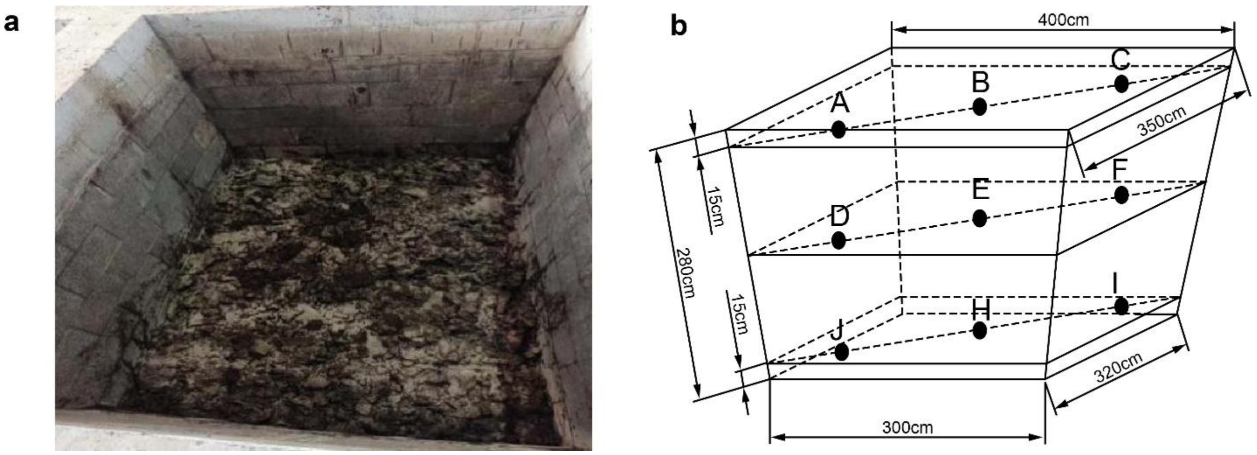

Figure S1. Fermentation cellar for compound flavor baijiu and sketch map of sampling during ultra-long fermentation.

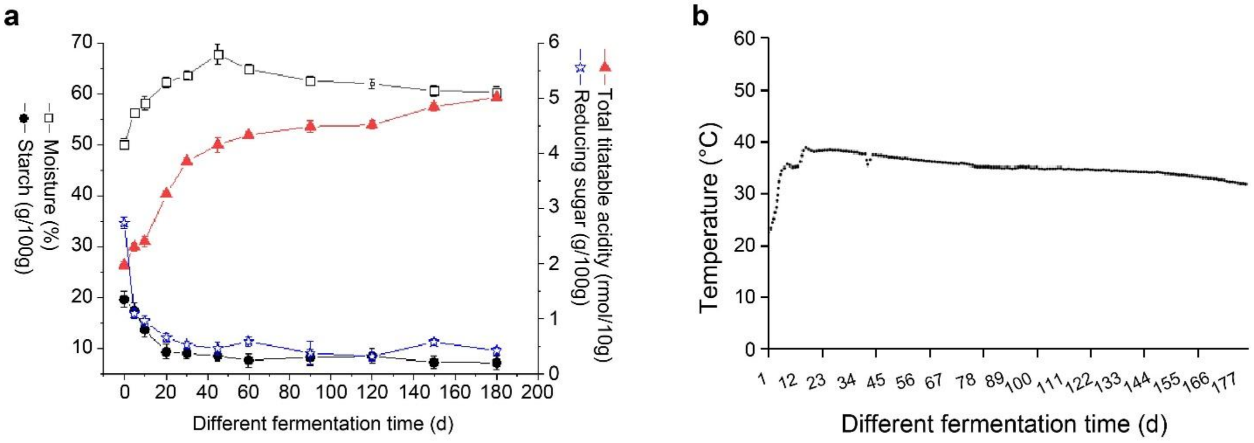

Figure S2. Changes in physicochemical parameters and temperature of fermented grains during the ultra-long fermentation process.

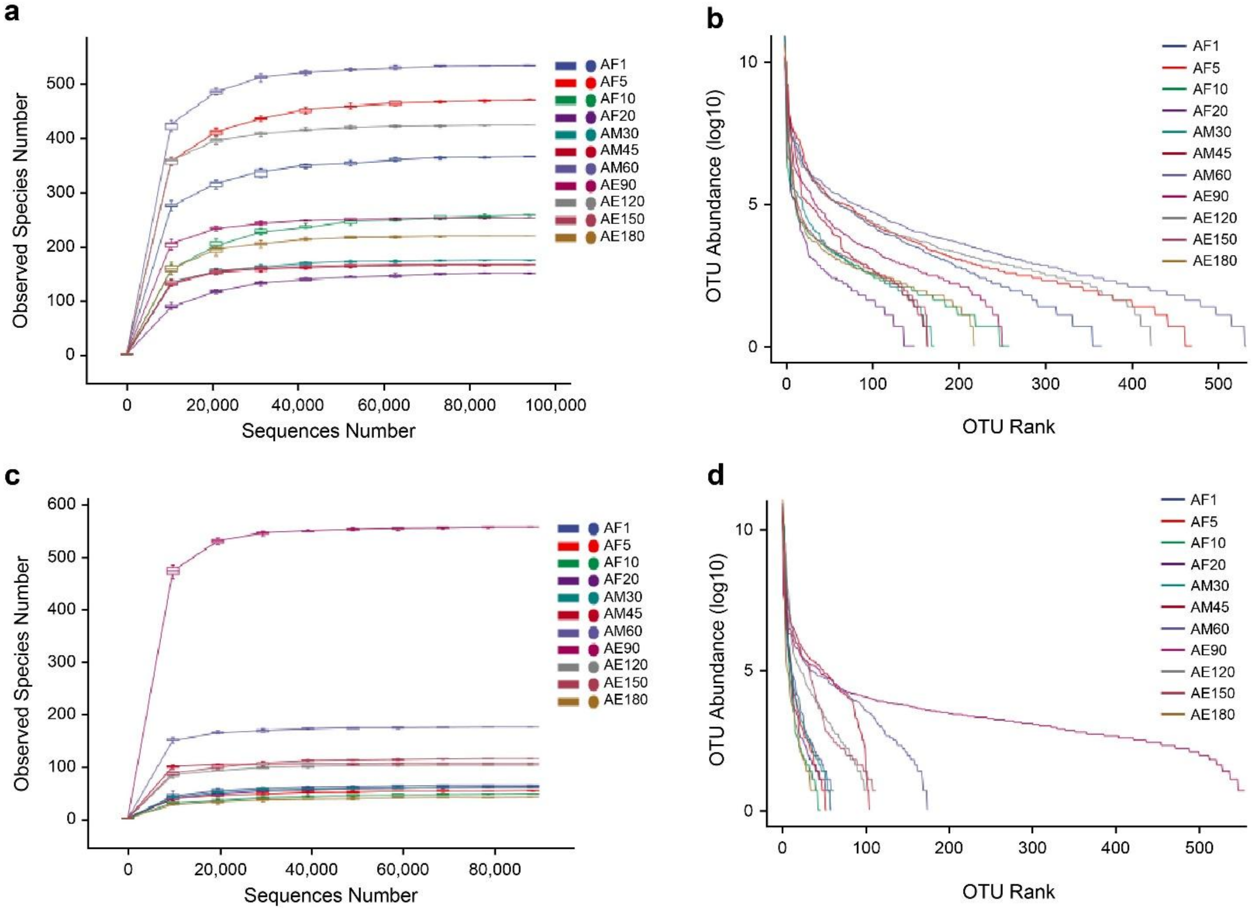

Figure S3. Rarefaction curves and Shannon curves of bacterial and fungal sequences and species from fermented grain samples.
